# Linking implementation science and policy: Process and tools for congressionally mandated implementation, evaluation, and reporting

**DOI:** 10.1111/1475-6773.14357

**Published:** 2024-07-24

**Authors:** Monica M. Matthieu, David A. Adkins, LaCinda Jones, Ciara M. Oliver, Jack H. Suarez, Barbara Johnson, Mona J. Ritchie

**Affiliations:** ^1^ HSR&D Center of Innovation Center for Mental Healthcare & Outcomes Research, Department of Veterans Affairs Medical Center Central Arkansas Veterans Healthcare System North Little Rock Arkansas USA; ^2^ School of Social Work Saint Louis University St. Louis Missouri USA; ^3^ VA Behavioral Health Quality Enhancement Research Initiative (Behavioral Health QUERI) Central Arkansas Veterans Healthcare System North Little Rock Arkansas USA; ^4^ VA Evidence‐based Policy Implementation Center Quality Enhancement Research Initiative (EPIC QUERI) Central Arkansas Veterans Healthcare System North Little Rock Arkansas USA; ^5^ Department of Psychiatry and Behavioral Sciences, College of Medicine University of Arkansas for Medical Sciences Little Rock Arkansas USA

**Keywords:** implementation science, legislation, policy analysis, QUERI, veterans, VHA

## Abstract

**Objective:**

To describe a process model for assisting partners in addressing requirements of legislation and review policy analysis, planning, and evaluation design processes and tools. Throughout its 25‐year history, the United States Department of Veterans Affairs (VA) Quality Enhancement Research Initiative (QUERI) program has been a forerunner in partnering with organizational leaders to improve health care. The Foundations of Evidence‐based Policymaking Act of 2018 provided new opportunities for QUERI and other implementation scientists to support federal agency leaders in implementing, evaluating, and reporting on congressionally mandated programs. Although implementation scientists have the skills to support partnered implementation and evaluation, these skills must be adapted for congressionally mandated projects as many scientists have limited experience in policy analysis and the intersection of data informing organizational policy, programs, and practices (i.e., evidence‐based policy).

**Data Sources and Study Setting:**

During the conduct of four congressionally mandated projects, our national VA QUERI team developed processes and tools to achieve the goals and aims of our VHA partners and to ensure our collective work and reporting met legislative requirements.

**Study Design:**

Our process model, program planning, and analysis tools were informed by an iterative process of refining and adapting the tools over a period of six years, spanning the years 2017 to 2023.

**Principal Findings:**

Work to support our partners was conducted across three phases: preparation and planning, conducting implementation and evaluation, and developing the congressionally mandated report. The processes and tools we developed within the context of mutually respectful and honest partnerships have been critical to our QUERI center's success in this area.

**Conclusions:**

Lessons we learned may help other scientists partnering in VA or other federal agencies to plan, conduct, and report on congressionally mandated projects.

**What is known on this topic**

- Congress holds the power and the purse strings for the entire federal government; legislation also drives which policies, programs, and practices federal agencies must implement, evaluate, and report to Congress.
- Analyzing legislation is both technical and challenging, requiring scientists to adapt their implementation, evaluation, and reporting skills to a new audience of executive‐level organizational leadership, policy makers, and Congress.
- Congressionally Mandated Reports require a macro‐level view to contextualize evaluation findings, align legislative and organizational priorities, and meet legislative requirements.

**What this study adds**

- Preparation of Congressionally Mandated Reports is best done in active, engaged collaboration between scientists and executive leadership.
- The process model supports implementation planning, program evaluation and legislative reporting requirements by identifying planning tools that can be leveraged to assist executives who are tasked with Congressionally Mandated Reports.
- The six‐step planning and preparation phase includes finding the legislation, reviewing it, drafting an outline, identifying the purpose and impacts, preparing a summary of the legislation, and creating a plan.

## 1 INTRODUCTION

The United States (U.S.) Department of Veterans Affairs (VA) Quality Enhancement Research Initiative (QUERI) has a unique history and role in promoting implementation and improvement science. Over the past 25 years, QUERI developed and maintained an intentional focus on system‐wide improvement of healthcare quality by encouraging scientists with relevant expertise to develop partnerships with healthcare executives (HCE), broadly defined herein as those who participate with the executive and management team, medical staff, and clinical leaders in the organization's decision‐making structures and processes across the Veterans Health Administration (VHA). Initially, QUERI funded condition‐specific research centers guided by Executive Committees composed of subject matter experts with diverse clinical and scientific backgrounds in those veterans' health conditions. QUERI centers were responsible for planning and conducting activities, including research, to ensure the rapid implementation and sustainment of evidence‐based practices and innovations.^1^, ^2^

Over time, QUERI implementation scientists began to partner with local‐, regional‐, and national‐level executives and clinical leaderships of a variety of healthcare programs to develop implementation strategies and study their use in large‐scale VHA initiatives.^3^, ^4^ They also provided consultation for the development of organizational policy and policy implementation, (e.g., VA's Uniform Mental Health Services Handbook).^5^, ^6^ In 2015, QUERI was reorganized to be more responsive to VHA's changing priorities and to place even greater emphasis on bidirectional partnerships with executives across all levels in VHA. In doing so, QUERI began to fund partnered evaluations that assess the impact, spread, and sustainability of policies, programs, and practices that support healthcare operations across the entire VHA.^7^

In 2018, Congress passed the Foundations for Evidence‐based Policymaking Act^8^ which was the genesis for modernizing research, policy, and evaluation activities across the entire federal government. It required federal agencies to focus staff and resources on evidence‐building, planning activities, and future needs assessments of evaluation capacity within and across their organizations. The passage of this Act resulted in exponential growth in the area of evidence‐based policy, including mandates for the implementation, evaluation, and reporting of findings related to new and ongoing programs and policies supported by federal funds. Not surprisingly, this law provided new opportunities for QUERI implementation scientists to support VHA executive‐level leadership in administrative operations and clinical programs. For example, to meet the requirements of the legislation directed to VHA, QUERI scientists often help with certain activities, such as: identifying critical differences between planned and actual implementation; identifying barriers to and facilitators of implementation; developing evaluation metrics; developing program logic models and evaluation plans; improving the implementation or evaluation process of existing programs; informing next steps such as assessing the feasibility and advisability of expanding or institutionalizing a new program; and preparing findings and recommendations that executive leaders can submit to Congress on behalf of VHA in response to legislative requirements.

Although implementation scientists possess skills needed to conduct research and quality improvement projects, these skills must be adapted to support executives in federal agencies who are faced with legislative requirements for program implementation and evaluation. The tasks required to support projects mandated by legislation, referred to herein as congressionally mandated projects, are different than those required for principal investigator (PI)‐initiated research projects; and the individuals responsible for those tasks may be different. (See Table 1 for a comparison.) For example, analyzing legislation required for congressionally mandated projects is both technical and challenging; many classically trained implementation scientists have limited experience in policy analysis. Additionally, many scientists have experience in initiating, planning, and conducting projects in partnership with executives at different levels within an organization. However, when executives in federal agencies who manage programs across local, regional, or national locations have primary responsibility for addressing the legislative requirements, the role of partnering with scientists in project development, implementation, and evaluation requires a different mindset. All scientists have the skills needed to produce reports and peer reviewed journal articles; yet, policy writing, such as writing briefings and reports for executive‐level leadership, policy makers, and Congress, and drafting evidence‐supported recommendations for an entire organization within Congressionally Mandated Reports (CMR), is different than academic writing. For example, a CMR is a formal report required by statute, prepared by a federal agency, and submitted to committees or subcommittees of the United States House of Representatives or Senate. Because the legislative requirements may vary, CMRs may require written descriptions, plans, findings from evaluations or studies, and reports describing notifications or actions taken by the federal agency.^9^ Policy writing, thus, requires an additional skillset. This skillset includes the ability to contextualize, for example, implementation plans, evaluation findings, descriptions of programs, or other notifications or actions (e.g., notifying Congress when metrics or measurement frameworks are developed) within the landscape of current and future legislation. The ability to contextualize helps to align legislative and organizational priorities and to assist agency executives in meeting the legislative requirements.^9^

**TABLE 1:** Comparison of tasks for research and congressionally mandated projects.

Implementation research project tasks	Congressionally mandated project tasks
**Responsibility for project**	**Responsibility for project**
PI proposes research idea and seeks funding.	Congress passes an organization‐specific law requiring implementation, evaluation, or reporting on a program and funds or requires the organization to fund the work.
PI ultimately responsible.	Healthcare executive (HCE) ultimately responsible; CMR Project Lead provides management.
**Project staffing**	**Project staffing**
PI responsible for hiring/contracting staff for the duration of funding.	CMR Project Lead identifies and selects internal staff to be detailed or hires new staff/contractors with HCE approval.
**Project planning**	**Project planning**
PI and Co‐I(s) plan the project focusing on implementation and evaluation of a specific innovation.^a^	Congress may dictate specific elements of the design, metrics, and outcomes of interest in the legislation. CMR Project Lead is responsible for project planning or hiring for planning; HCE approves all plans. IS may provide project planning services or provide consultation on existing project plans.
**Conducting the project**	**Conducting the project**
PI or Co‐I(s) engage and recruit sites.	HCE or CMR Project Lead may engage or recruit sites. IS may interact with sites as needed.
PI, Co‐I(s), and their team implement innovation^a^, collect, and analyze data.	CMR Project Lead supports staff to implement innovation^a^, supports staff or contractors to collect and analyze data. IS may implement some or all aspects of innovation and collect and analyze some or all of the data.
**Dissemination of findings**	**Dissemination of findings**
PI reports on findings.	HCE is responsible for developing and submitting all reports. CMR Project Lead may draft some or all of required reports and is responsible for drafting recommendations for the organization. IS ensures the data quality and integrity for findings; may draft/edit some or all of the reports or recommendations.
PI or Co‐I(s) present findings to internal or external audiences.	HCE presents briefings to Congress and organizational leadership; CMR Project Lead may be asked to participate in legislative briefings. IS may present findings if authorized by CMR Project Lead and approved by HCE.
PI produce articles for publication	IS may publish findings if authorized by CMR Project Lead and approved by HCE.

Abbreviations: CMR, congressionally mandated report; Co‐Is, co‐investigators; HCE, healthcare executive; IS, implementation scientists; PI, principal investigator.

^a^ An innovation is a generic term to denote a specific practice, program, or policy.

During the years 2017 to 2023, our team of QUERI scientists conducted implementation, evaluation, and reporting projects in partnership with VHA executives who provided oversight and leadership to either the administrative or clinical operations of national‐level VHA healthcare programs in response to four different congressional mandates. Each project required a CMR. Lessons we learned conducting these projects in VHA may benefit other scientists partnering with executives in VHA or other federal agencies to support evidence‐based policy implementation and evaluation. In this paper, we describe our methodology and a process model consisting of three broad phases for supporting such projects: preparation and planning, conducting the work, and creating a CMR. The objectives of this paper are: (1) to describe the process model for assisting partners to address the requirements of legislation and (2) to review helpful policy analysis, planning, and evaluation design processes and tools.

## 2 METHODS

### 2.1 The team

For this work developing our phased methodology, our team was associated with two QUERI centers, the Behavioral Health QUERI and the Evidence, Policy, and Implementation Center (EPIC) QUERI. This team was led by two doctoral‐level social work researchers with over 20 years each of clinical, research, and evaluation experience in implementation science and applying systems thinking to improve care of veterans in VA healthcare settings; a master's‐level healthcare administrator with over 30 years of experience in military healthcare settings; and a master's‐ level social worker with additional training via a congressional fellowship in healthcare policy, analysis, healthcare law, and legislative activity expertise. Other team members included technical writers, analysts, project coordinators, administrative assistants, VHA subject matter experts, and scientists who supported the teams on specific projects requiring CMRs.

### 2.2 Our partners

The partners for all our CMR projects were national‐level VHA HCE who were assigned and provided oversight to support the delivery of the CMR. Our day‐to‐day partners were the leaders of the specific programs or organizational unit that most closely aligned with the focus of the legislation and who were tasked with managing the team to address the legislative requirements. Further, these CMR Project Leads were subject matter experts responsible for their respective program and for the CMR project that implemented the new legislative requirements associated with or related to their program.

### 2.3 The projects

Our team worked on four projects that required CMRs and consulted with clinical leadership on one other project that did not require a CMR. For all projects, we arranged a consultation call between the lead QUERI scientist, a policy analyst, the partners, and other relevant individuals. The purpose of these calls included discussion about the legislation, identification of any requirements and timelines for reporting, opportunities to join the efforts long‐term, funding, and any other desired goals and deliverables. As of the end of 2023, three of four CMRs were submitted, with one project spanning five years until the CMR is due.

### 2.4 The development process

In working with partners, we first identified their goals and needs for the congressionally mandated project, which may include one or all the following: implementation, evaluation, and reporting. This needs assessment led our team to identify useful processes and tools, many of which we used previously in the development of grants and projects. Over time, we iteratively developed, refined, and adapted these processes and tools to achieve our partners' aims and goals and to ensure that our collective work and reporting meets legislative requirements.

## 3 PROCESSES AND TOOLS FOR SUPPORTING CONGRESSIONALLY MANDATED PROJECTS

The first phase of our three‐phase process model focused on preparation and planning activities, including engaging with our partners. The second phase focused on activities to support partners' requirements for implementing and evaluating practices, programs, or policies. The third phase focused on reporting activities to support the creation of the CMR, including mapping the outcomes and impacts of the evaluation to legislative requirements and working collaboratively with partners to develop recommendations for Congress. Below, we describe activities that we conducted with HCE and CMR project leads, hereafter collectively referred to as our partners, or as executives and leads singularly, and examples of how these activities supported our projects.

### 3.1 Phase one: Preparation and planning

During this first phase, we started by actively engaging with our partners. Engagement activities were always important because the partnership may be new and may include contractors or other team members who do not know about the program being implemented, did not participate in matching the program components to address legislative requirements, or could not establish a data collection protocol aligned with the legislation at the time of program implementation. Additionally, scientists and many of the partners' staff may know nothing about each other's roles, responsibilities, skills, and experience. Attention to establishing bidirectional communication with the responsible HCE at the outset set the foundation for identifying the expectations that guided and supported the project through to completion.^10^ For example, in one project, our team was invited to provide consultation after our partners had already established a plan for implementation and evaluation. Our early efforts to engage with partners, understand their needs and concerns, and foster bidirectional communication resulted in an expansion of the evaluation plan to include needed elements.

**FIGURE 1:**
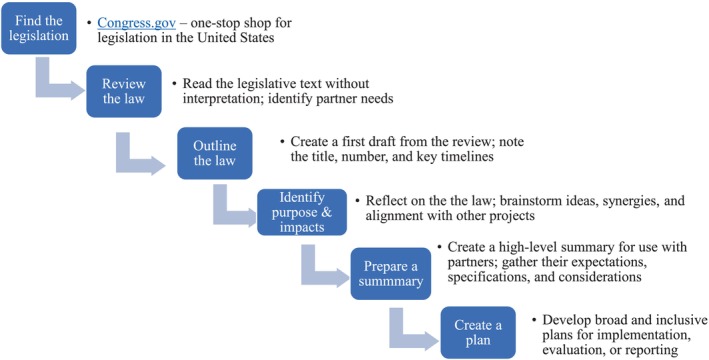
Phase one of the process model: Six steps in the preparation and planning process.

We also found that having a thorough understanding of the legislation and its requirements was essential for supporting our work. Reviewing the legislation allowed us to gain a greater appreciation and knowledge of the text of the bill to develop ideas on the purpose, requirements, and potential impact and to identify relevant timelines for reporting. This detailed review also prepared us for mapping legislative requirements to an initial program concept and for identifying potential implementation and evaluation methods. As we worked on congressionally mandated projects, we developed a preparation and planning process for fostering collaboration with our partners and preparing for the work that needed to be done to achieve their goals. In the first phase, there are a series of six steps; we use the process model to identify the requirements of the legislation and aid in the selection of tools needed to support our work, inform products that our partners may need, or to plan deliverables such as manuscripts, reports, and information to be used by executives for congressional testimony, dissemination, and reporting to other interested parties. Below, we describe the phase 1 steps (see Figure 1).

#### 3.1.1 Steps in the preparation and planning process

##### Step 1: Find the legislation

The website, Congress.gov, is the authoritative archive for the U.S. legislative process. We review the summary of the law, the Congressional Budget Score cost‐estimate for implementing the law, the legislative committee's analysis, if available, for context on the development of the law, and the history of the bill.

##### Step 2: Review the law

We review the entire legislative text that our partners have identified without applying any interpretation to understand the goals, aims, and intent of the law. Following this cursory review of the entirety to gain an understanding of their needs, we then focus specifically on the section of the legislation for which our partners have responsibility.

##### Step 3: Draft an outline of the law

Next, we take notes on key aspects of their section of the law, paying particular attention to numbers and dates. We then draft an outline which includes an overview of the law's intent and key timelines. These timelines establish deadlines for key deliverables, such as CMRs.

##### Step 4: Identify the law's purpose and potential impacts

Then, we reflect on what we have learned after analyzing the legislative text by asking questions about its purpose and what impacts we think it will have (see Table 2 for guiding questions). These early ideas on the potential impacts aid in developing recommendations which will be discussed later. We brainstorm ideas, synergies, and alignment with other projects by adapting some of the tools, as described in steps five and six, that we developed for use in other projects. For example, in our initial CMR project, the success of existing employees in engaging and supporting access to care initiatives important to our organization led to a second CMR project. In this second project, we built a team collaborating with leading researchers in the area, adapting previously tested data collection tools, metrics, and processes, and rapidly expanding our data analytic capabilities to build employee performance dashboards that summarize and visualize workload across various settings of care. Such dashboards remain critical for ongoing national program monitoring as the project subsequently expanded.

**TABLE 2:** Step 4: Key considerations when reviewing legislation and partnering on implementation, evaluation, and reporting projects.

✓	*What is the purpose of the law?*
	Does the law address a specific problem or issue?
	What is the magnitude, reach and distribution of benefit and burden, including impact on risk and protective factors, quality of life, morbidity, and mortality? Are there gaps in the data/evidence‐base?
	Who is responsible for administering the law?
✓	*What are the requirements of the law?*
	Does the law have any requirements for implementation, evaluation, or reporting?
	Does the law require a new design, modification, continuation or review of an existing program, policy, or practice?
	Does the program, policy, or practice need to be implemented or evaluated according to specific legislative requirements?
	What key activities need to be completed during policy implementation or evaluation?
	What inputs and resources are required to implement or evaluate the policy? Are all these inputs and resources available?
	Does the law come with appropriations or is it an unfunded mandate?
✓	*What is the potential impact of the law?*
	What are the objectives? What are the short, intermediate, and long‐term outcomes?
	What population(s) will benefit? How much? When? What population(s) will be impacted? How much? When?
	What might be the unintended positive and negative consequences of the law?
	Will the law impact health disparities/health equity? How?
	What is the public health impact of the law? Is the law value‐added?

##### Step 5: Prepare a summary

In consultation with partners, we then create a high‐level, one‐ or two‐page summary of the law in outline format which can be used to educate our partners or new team members. During the consultation process, we also gather information about their expectations, specifications, and considerations. For one project that did not require a CMR, we provided a scientific review‐focused consultation for clinical leadership in which we reviewed this summary. Based on their history with the program, implementation, and ongoing monitoring plans, they had decided that an evaluation was helpful but not necessary to meet legislative requirements. Our consultation provided much needed reassurance and support of their planned direction.

##### Step 6: Create a plan

To begin the planning process, we create a detailed, three‐to‐ten‐page outline of the law, including all relevant text. We add ideas generated in the previous steps, and we append these with questions or comments received from our partners. We may also invite members of our implementation or evaluation teams, comprised of qualitative and quantitative analysts, measurement, implementation, and other subject matter experts, to consultations with our partners. This broad and inclusive process can result in feedback on key terms needing definition by legal or legislative staff, rules and regulations needing to be codified and promulgated for the federal government in the design of the program, policy, or practice, as well as alternative methods or designs to be considered that arise from obstacles identified in early implementation and evaluation planning. In one CMR project, the legislative, policy, and communication representatives from the organization initially used this detailed outline to gather information about regulatory requirements. Building on our partners ideas, our team added initial ideas for program design and required metrics. This resulted in the use of an evidence‐based and subject matter expert‐informed process to select and review measures with VHA scientists and clinicians and to share the final list of metrics with legislatively required internal and external stakeholders.

Also during this step, we confirm the exact language of the text that explicitly documents a clear need to develop plans for implementation and evaluation of a new or existing program, policy, or practice. We document all specific details of the reporting requirements and calculate the timelines, paying careful attention to the language; for example, 18 months from program start versus 18 months from the date legislation is passed can vastly accelerate the project. In one of our CMR projects, the start date of the legislation was required to calculate the timeline until the due date of the CMR. For another, the legislation required the report years later using the program start date, which was a moving target until the organization aligned the completion of regulatory, compliance, and communication plans to officially announce the start date. From this start date decision, all timelines were adjusted. Overall, for CMR projects, the time allotted by the legislation and this timeline calculation informs not only the timeline for reporting, but also design decisions and implementation planning for new programs or policy startups.

#### 3.1.2 Other helpful tools

During this first phase, we also employed planning tools we adapted from our work on investigator‐initiated projects. In this section, we provide an overview of the tools and offer examples in applying them from our CMR projects, outlining differences as appropriate.

##### Scope of work

For a large complex project, we develop a Scope of Work (SOW), which is a written document that outlines the work that must be accomplished in the required timeframe and clearly delineates the roles, lanes of effort, and deliverables of the various entities involved in the project. Such a planning tool is especially helpful for congressionally mandated projects in which the work may be divided between (1) HCE and their team, including other staff hired, detailed, or contracted to the project, and (2) scientists' implementation and evaluation teams. While potentially broad in scope, for our team, the purpose of the SOW was to specifically identify the implementation and evaluation intersections and potential overlaps of roles, responsibilities, and deliverables. Thus, we provided partners and other team members who were new to evaluation or implementation with our best estimation of the resources, including staffing, technology, materials, data, and processes it will take to accomplish the aims of the project. This written SOW helped us, and our partners, focus on our designated roles in implementation and evaluation processes and identify any gaps. Although not necessary for every project, the SOW for one of our CMR projects was most helpful when we identified a need for specialized expertise that could not be obtained from within the organization. Identifying it early allowed our partners to plan time and resources to hire contractors. The SOW we developed was later used to provide technical requirements needed for the contracting process.

##### Program evaluation designs and plans

As implementation scientists, we develop evaluation plans when we are preparing proposals to apply for funding. All scientists are trained to create such plans. Additionally, QUERI has developed a number of helpful tools to support the design of organizational policy or program evaluations.^11^, ^12^, ^13^, ^14^ Congressionally mandated projects may or may not require an evaluation. However, some partners request an evaluation focused on the legislative requirements to include other projects or to support evaluation of broader policies; and they ask us to design, or help them design, an evaluation plan prior to conducting the project.

In preparing an evaluation plan for congressionally mandated projects, we use the process model described earlier to obtain insight into the core components and activities of the specific program, policy, or practice that needs to be evaluated. Similar to research proposals, we identify potential data, methods, metrics, key performance indicators, and measures of fidelity for assessing whether the core components of the program were implemented as planned. Being thoughtful and cognizant of preferred communication styles, we work closely with key partners and team members involved in the larger project, beyond the evaluation, to negotiate optimal methods and deadlines for obtaining information and feedback on the evaluation plan for briefings and presentations. When evaluation planning is unknown to our partners, we provide education and coaching to help them. In all our projects, our team goal is to continually educate our partners. The outcome of this investment has been that in many projects, our partners and their staff have exhibited increasing skills in implementation and evaluation planning as we worked with them over time by building capacity for future planning and reporting efforts.

As one example of using education to build our partner's capacity, a CMR project lead for a long‐term project was detailed for a few months to another of our CMR projects that had a gap in staffing and was under a short timeline to complete the report. Following a briefing we provided on developing the CMR to their new team, we continued to support their learning in our weekly project meetings with key tips offered along the way in preparing the CMR. Upon the conclusion of their detail, we discussed their experience with a CMR project and decided to intentionally take lessons learned around key metrics and writing recommendations from one project to the other given we have a few years until our long‐term CMR project concludes.

Returning to evaluation planning, we remain careful to identify the leadership (e.g., HCE, CMR project lead, or other subject matter experts) ultimately responsible for approving the plan and monitoring program implementation, as well as the timeline for when the program may start. In our experience, the development and approval of an evaluation plan for a new congressionally mandated program takes six to nine months, as nearly every aspect of the program needs to be delineated in order to create the final evaluation plan. Simultaneously developing the program and creating the evaluation plan can have many benefits; however, program decisions regarding cohorts, timing, rules, regulations, metrics, and performance measures can change the evaluation design, measures, and data collection systems making this process rather complex.

Finally, we may also work with our partners on planning who will conduct the evaluation. There are several options. Plan developers may become the evaluation team. However, other individuals, teams, or contractors might develop or conduct different aspects of the evaluation based on what can or cannot be outsourced easily. When all data collection and analysis will be outsourced, the role of scientists may be restricted to designing and obtaining approval for an evaluation plan or returning after the analysis to write the report. These latter two options are often short‐term, rapid projects with a niche for scientists, especially when they are not retained to support the progression of the CMR project from conceptualization to reporting.

##### Logic models

Of late, we have been increasingly asked for more planning documents up front and much earlier in the planning phases of a new project. Logic models, described as “graphic depictions of the shared relationships among various elements of a program or study,” have been used in research for a variety of purposes, including planning.^15^ Because programs, polices, and practices may involve hierarchical complexity^16^ or concurrent evaluations of different aspects of the same policy that is not easily represented,^17^ logic models may provide a way to see the connections between different implementation and evaluation elements, including inputs, outputs, strategies, and outcomes, specifically for programs and practices in healthcare delivery settings.^15^ Logic models can be complex. As part of the design phase in one project, we developed program logic models to visualize the evaluation design with prospective measures, metrics, and outcomes to share with our partners. Consistent with the experience of others,^10^, ^18^ our partners found these models most helpful when we used lay language without research jargon and when the information was presented with brevity and clarity. When logic models have been created in this manner, our partners have been able to integrate them into program briefings and other materials they produce and share broadly, including with Congress.

### 3.2 Phase two: Conducting the work of implementation and evaluation

As noted earlier, there are three broad phases in our process model for supporting CMR projects. While the first phase focused on all the planning, the second phase is the actual conduct of the planned work. In this phase, scientists may be asked to conduct a range of activities to include building and managing teams of subject matter experts, staff, or contractors to support partners' requirements for implementing and evaluating the required practices, programs, or policies. In one CMR project our team was responsible for the implementation, evaluation and reporting, while in three others we are only tasked with the evaluation and reporting requirements. Once mobilized, the plans guide the work to unfold as expected for most scientists.

However, in this phase, there are a few differences in implementation and evaluation of CMR projects compared with investigator‐initiated research. One major difference is related to who has responsibility for the project. As noted earlier in Table 1, the ultimate responsibility for the CMR project rests with the HCE who provides oversight to the CMR project lead, whereas in research projects, the PI has this responsibility. Another difference is that CMR projects are fluid. In our projects, changes have sweeping impacts that can ripple across programs in the federal government, thus, we learned to respond positively and creatively to change. Our change‐oriented mindset allows us to mobilize real‐world solutions to these fluctuations when they occur. In one project, Congress passed an appropriation that allocated a significant amount of funding that arrived in the last years of a project. This influx of funding was tied to new legislative requirements to expand the program in specific ways. With so little time left in the project, we opted to first identify capacity building activities that would be helpful to existing sites and then spread the remaining resources beyond the current sites involved in the project to quickly recruiting and onboarding new sites. As a result, the size of the program tripled. As implementation scientists with a systems‐level view and experience doing projects, we can often see how best to optimize resources and build out an existing project, even when the scope of the project at the beginning may be extremely narrow.

### 3.3 Phase three: Developing the congressionally mandated report (CMR)

A CMR is a written report required by legislation to be submitted by federal agencies to the Senate, the House of Representatives, or to congressional committees or subcommittees.^19^ The report is expected to respond to the legislation based on the specific requirements. If an evaluation is required, the report may contain an overview of the methods, the overall evaluation findings, and any specific data elements as required in the CMR.

When the legislation requires a CMR, we develop a CMR template during the initial planning process of phase one using the process model described earlier. This template includes the required reporting elements from the legislation laying in the evaluation plan elements. When conducting the evaluation in phase two, we populate the CMR template with relevant data and results as they are available, often coinciding with annual, or other reviews of the program or in response to requests for results by funders, partners, Congress. Creating the template and updating it along the way ensures that we have a systematic way to collect, analyze, and summarize data that may later be included in the actual report.

Later, nearing the end of the project and well prior to the CMR due date, we create a draft of the CMR. The format of the CMR is akin to most evaluation reports, with a few notable adjustments. The first part of the report includes a summary of the legislation, timeline, and requirements. Rarely is a literature review included, however citations to justify design selections may be appropriate. Following this, we outline the methods in a brief format like a structured abstract. While the HCE leading the project will typically provide the most up to date template that is required for submission of the CMR, we first organize our initial draft of the results section using the legislative requirements as section headers. For each subsection, we briefly outline the methods, data sources, and results of the evaluation. Then, we draft answers to direct questions that were asked in the legislation and incorporate only the data or information needed to answer the specific question posed by the legislation for impacts.

Using this initial draft, we then provide a briefing to our partners. To ensure scientific and data integrity, we identify data and data interpretations that should not be changed in the editing process. We advise our partners and their team members on what data and information they might include in the report and offer considerations on the potential advantages and disadvantages of including or excluding other data and information.

Once the briefing has concluded, the initial draft is provided to the partners who are primarily responsible for writing and editing the CMR. We edit and review material as requested by and in collaboration with our partners. Having developed a mutually respectful, honest, and clear partnership at the beginning helped us build trust. This trust has been honed over time and is based on professionalism, consistency, and integrity. Moreover, using this iterative and collaborative writing and editing process ensures we remain focused on helping our partners refine the CMR. In comparison with the initial draft, this middle stage of writing and editing the report seeks to contextualize the data from the evaluation results into the policy landscape. This landscape can include current or ongoing legislative requirements, organizational priorities, strategic plans, and learning agendas, as well as the programs' effectiveness or other required evaluation elements.

The final step in CMR preparation is the development of recommendations for Congress; this process can be daunting. These recommendations are more than just the implications of the study required in research reports. Although we draft CMR recommendations broadly with reference to potential future program, policy, and research or evaluation, they also suggest actions the organization or Congress can take as a result of the findings documented in the CMR.^9^ Our partners, with our support if requested, edit these recommendations and submit this draft to the responsible executive's office. The CMR is then finalized using an iterative process of review and refinement. The responsible executive's office submits it to various review and approvers along the way up to the highest official in the organization, which in our case, is the Secretary of Veterans Affairs, and finally to Congress.

In summary, CMRs take time and concerted attention; we allocate up to six months for the drafting of a CMR. This does not include two to three additional months for the review, editing, refinement, and vetting process with our partners, nor the clearance process. The clearance process, which ranges from two weeks to three or more months, includes technical, legal, regulatory, and compliance reviews and edits by executive‐level leadership who must approve the report. At the end of this process, the report is final and, thus, “cleared.”

We also continue to support our partners well after the submission of the CMR to Congress because they may have other questions needing additional analyses or responses based on the interest, requests for technical clarification, or feedback on the report from Congress. We maintain a partner‐focused mindset from beginning to end and welcome the ability to assist; however, we do communicate to our partners that when the project and funding ends, our project staff will need to move on to other funded projects. The responsible scientist remains available for typically 3–6 months to assist the partners as questions arise, to review executive‐level responses to Congressional inquiry and to consult on the scientific presentation of the data. Lastly, for dissemination, we prioritize our partners presenting the CMR and representing the project over our own team. Should we present on behalf of the project, we consult with our partners on the content and secure their approval for the presentation.

## 4 DISCUSSION

For the past 25 years, VHA's QUERI program has focused on improving the health care of veterans while leading advancements in implementation science and supporting VA's transformation to a learning health system. Over these years, QUERI has also been developing a cadre of implementation scientists with the knowledge and skills to support VHA executives in their efforts to improve the health care provided to veterans. Increasingly, legislation is driving some of the policies, programs, and practices VA must implement, evaluate, and report to Congress. In addition to creating Evidence‐based Policy Evaluation Centers to expand VA's policy evaluation capacity, QUERI encourages its implementation scientists to partner with clinical and operational leaders in support of policy initiatives. Although these scientists are uniquely poised to provide such support, their skills need to be adapted to the policy context.

Based on our experience with four congressionally mandated projects, in this paper, we describe some of the processes and tools that have been helpful in adapting our skills for such projects. Specifically, we described the three phases of a process model for supporting such projects and a six‐step process model that can be used during the preparation and planning phase for identifying legislative requirements, selecting tools, informing products our partners needed, and planning deliverables for use by executive‐level leadership. We also described how we have adapted other processes and how we developed compulsory reports and recommendations in partnership with HCE. Lessons we have learned and described in this paper may help other implementation scientists and even HCE and their teams in other federal agencies to plan, conduct, and report on congressionally mandated projects.

As QUERI improvement and implementation scientists, our research and evaluation work aims to have real‐world impact. Knowledge from how a bill becomes a law to the use of a process model for how to discern and use policy analysis, planning, and design tools for congressionally mandated implementation, evaluation, and reporting projects in VHA has been critical to our QUERI team's success in this area. Armed with new processes and tools, QUERI improvement and implementation scientists have the skills and ability to lead in this area of congressionally mandated implementation and evaluation projects for years to come.
